# Supplementary material for: Molecular evolution and the decline of purifying selection with age
Source: Nat Commun. 2021 May 11;12:2657. doi: 10.1038/s41467-021-22981-9 (PMC8113359; doi:10.1038/s41467-021-22981-9)
Supplement: Supplementary file 1 — Supplementary Information [file 41467_2021_22981_MOESM1_ESM.pdf]

Supplementary Information for

"Molecular Evolution and the Decline of Purifying Selection with Age"

by C. Cheng and M. Kirkpatrick

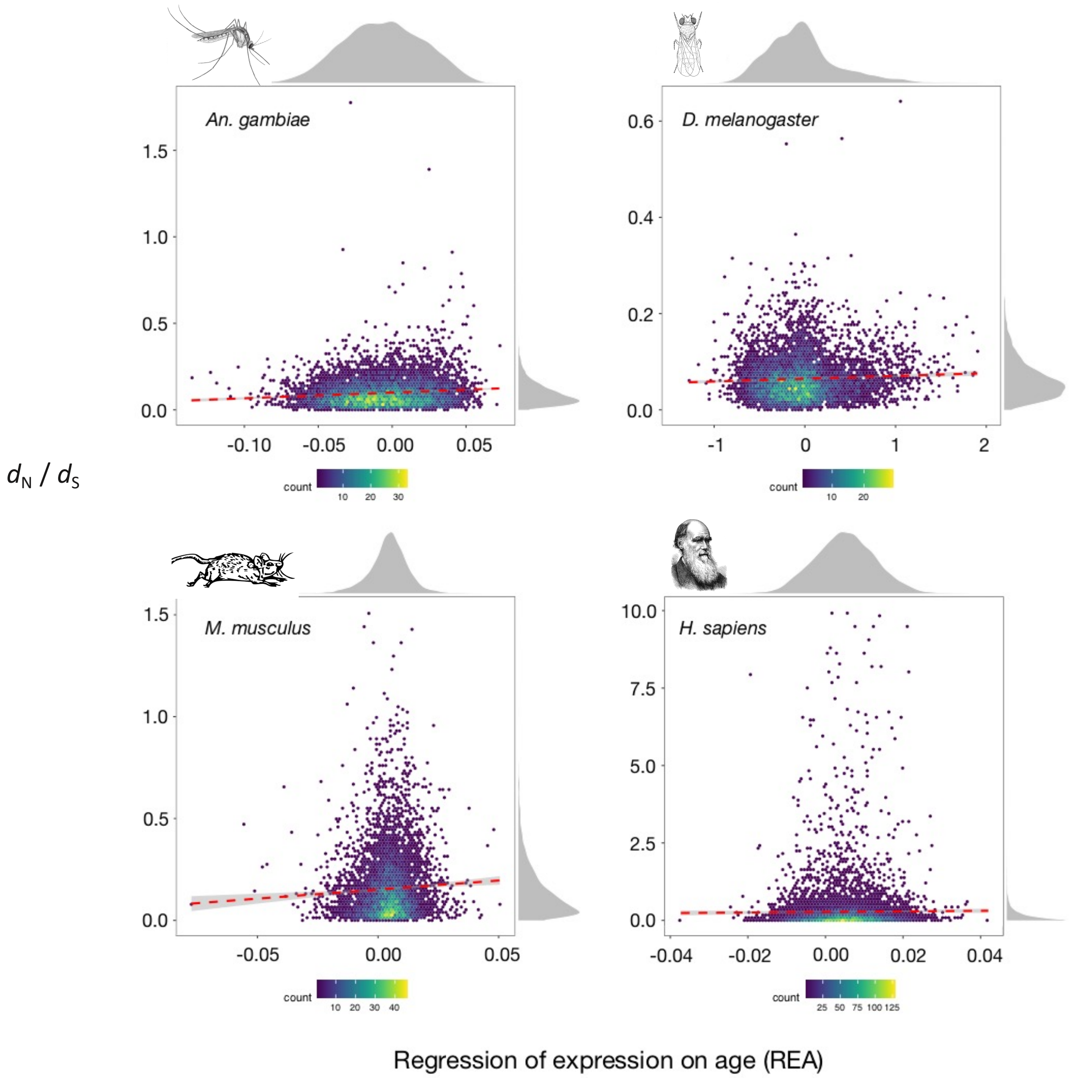

Supplemental Fig. S1: Late genes fix nonsynonymous mutations at higher rates. Genes expressed later in life fix nonsynonymous mutations at a significantly higher relative rates than those expressed early, as measured by the d_N/d_S ratio, in mosquitoes, flies, mice, and humans. The dashed lines are least squares regressions, and the grey regions show the approximate 95% confidence intervals for the regressions. See also Fig. 1 and Table S1.

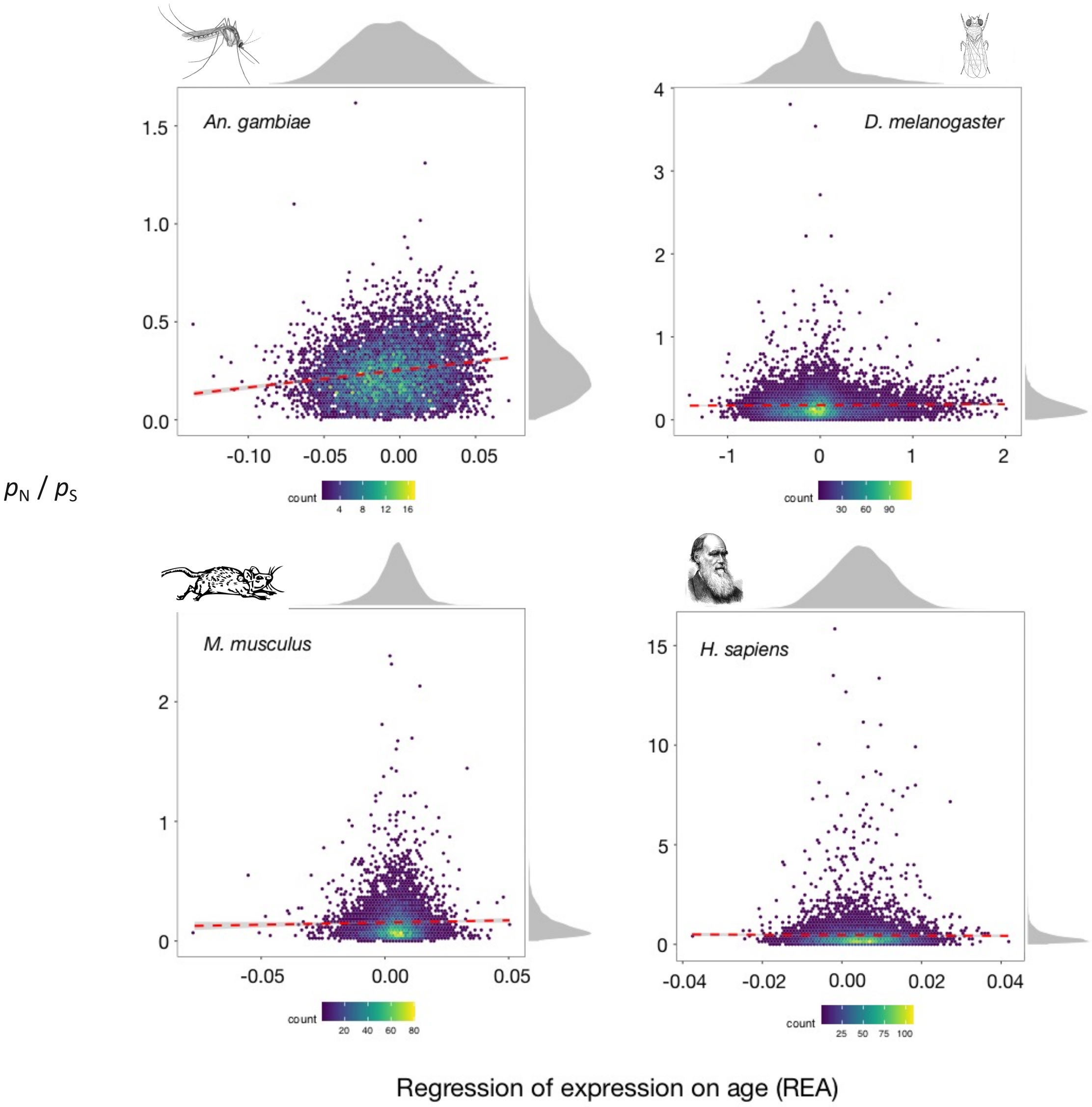

Supplemental Fig. S2: Late genes are more polymorphic for nonsynonymous mutations. Genes that are more highly expressed late in life are significantly more polymorphic for nonsynonymous mutations than are genes expressed early in all four species. Nonsynonymous polymorphism is measured here as the fraction of sites in a gene that segregate for nonsynonymous alleles (p_N) relative to the fraction segregating for synonymous alleles (p_S). The dashed lines are least squares regressions, and the grey regions show the approximate 95% confidence intervals for the regressions. See also Fig. 2 and Table S1.

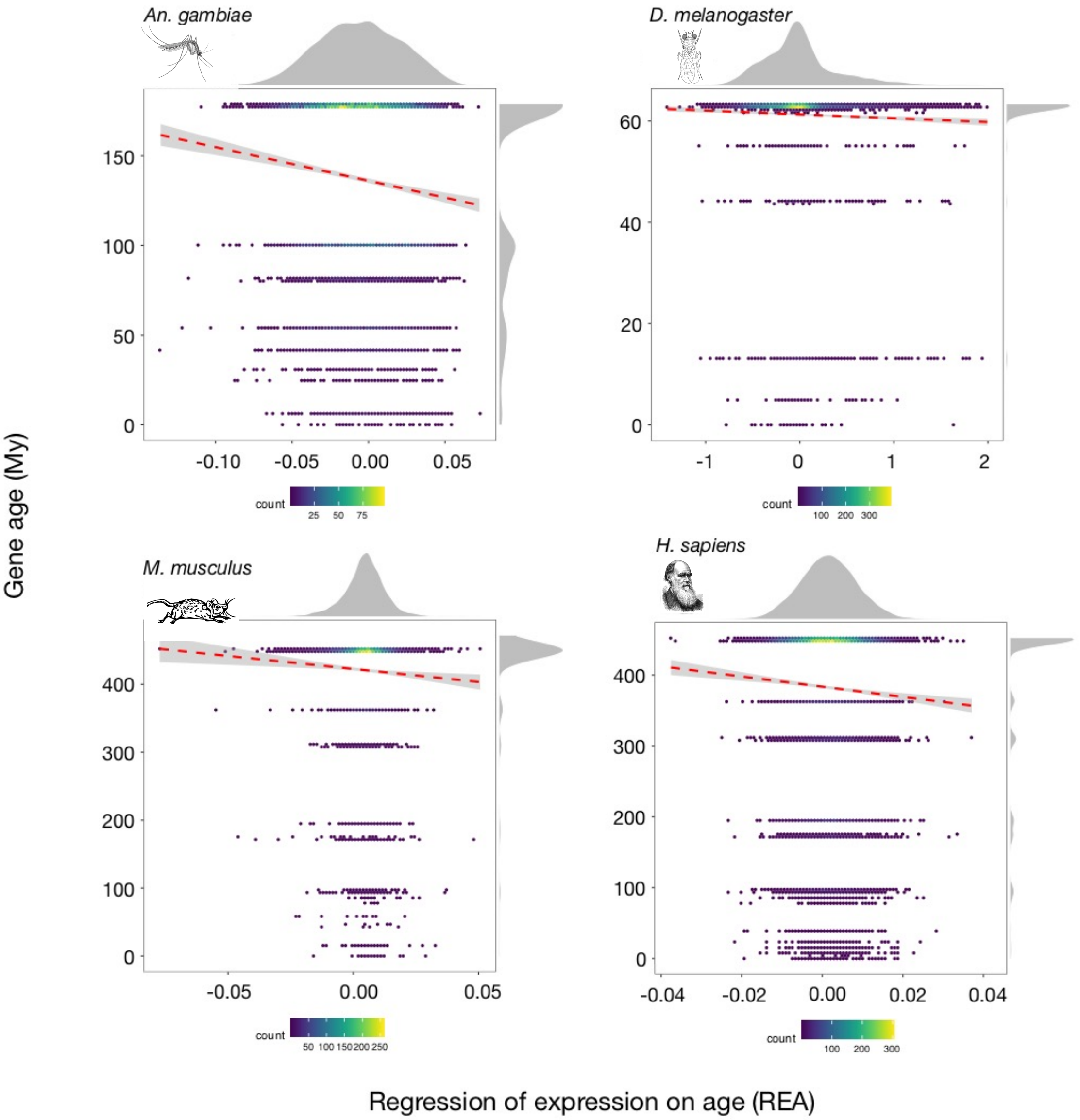

Supplemental Fig. S3: Late genes are younger. Genes expressed later in life originated significantly more recently than those expressed early in all four species. The dashed lines are least squares regressions, and the grey regions show the approximate 95% confidence intervals for the regressions. See also Fig. 3 and Table S1.

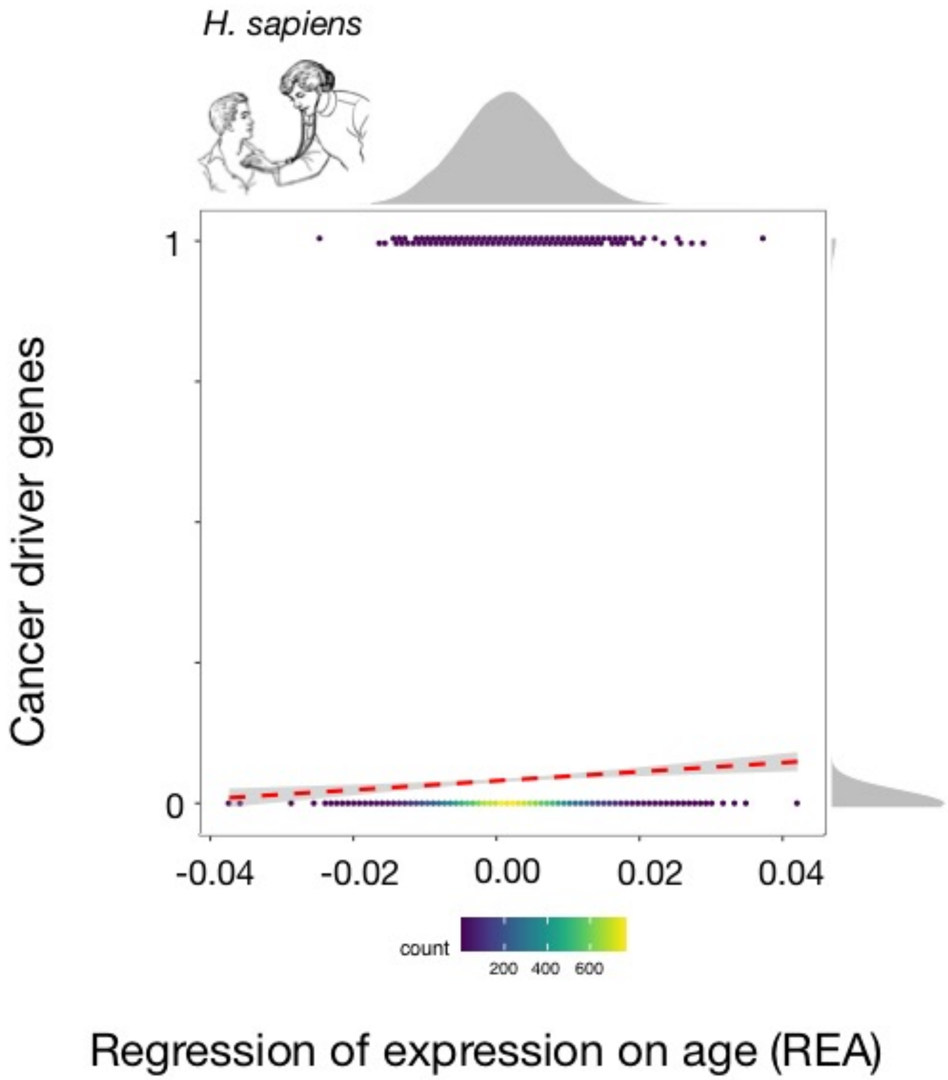

Supplemental Fig. S4: Late genes are more likely to be driver genes for adult cancers in humans. The probability that a gene is associated with adult cancers is correlated with its age of expression in humans. The dashed line is the least squares regression, and the grey region shows the approximate 95% confidence interval for the regression. See also Fig. 4 and Table S1.

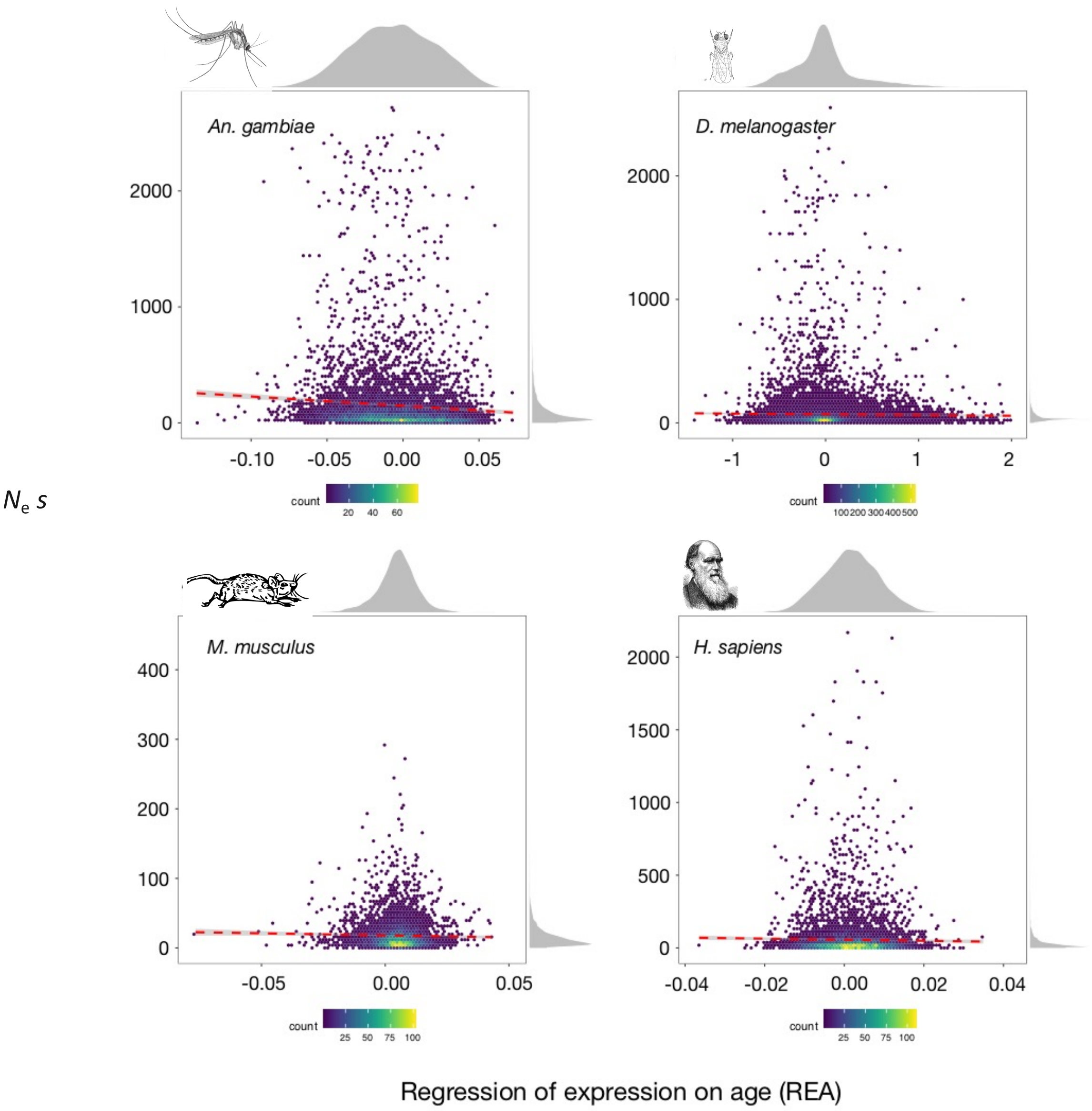

Supplemental Fig. S5: Selection on late genes is weaker. The relative strength of selection is significantly weaker in late-expressed genes than early-expressed genes in mosquitoes, mice, and humans. The estimates of $N_e s$ are based on a highly simplified model that assumes beneficial mutations have fitness effect *s* and deleterious mutations fitness –*s*. The dashed lines are least squares regressions, and the grey regions show the approximate 95% confidence intervals for the regressions. See also Fig. 5 and Table S1.

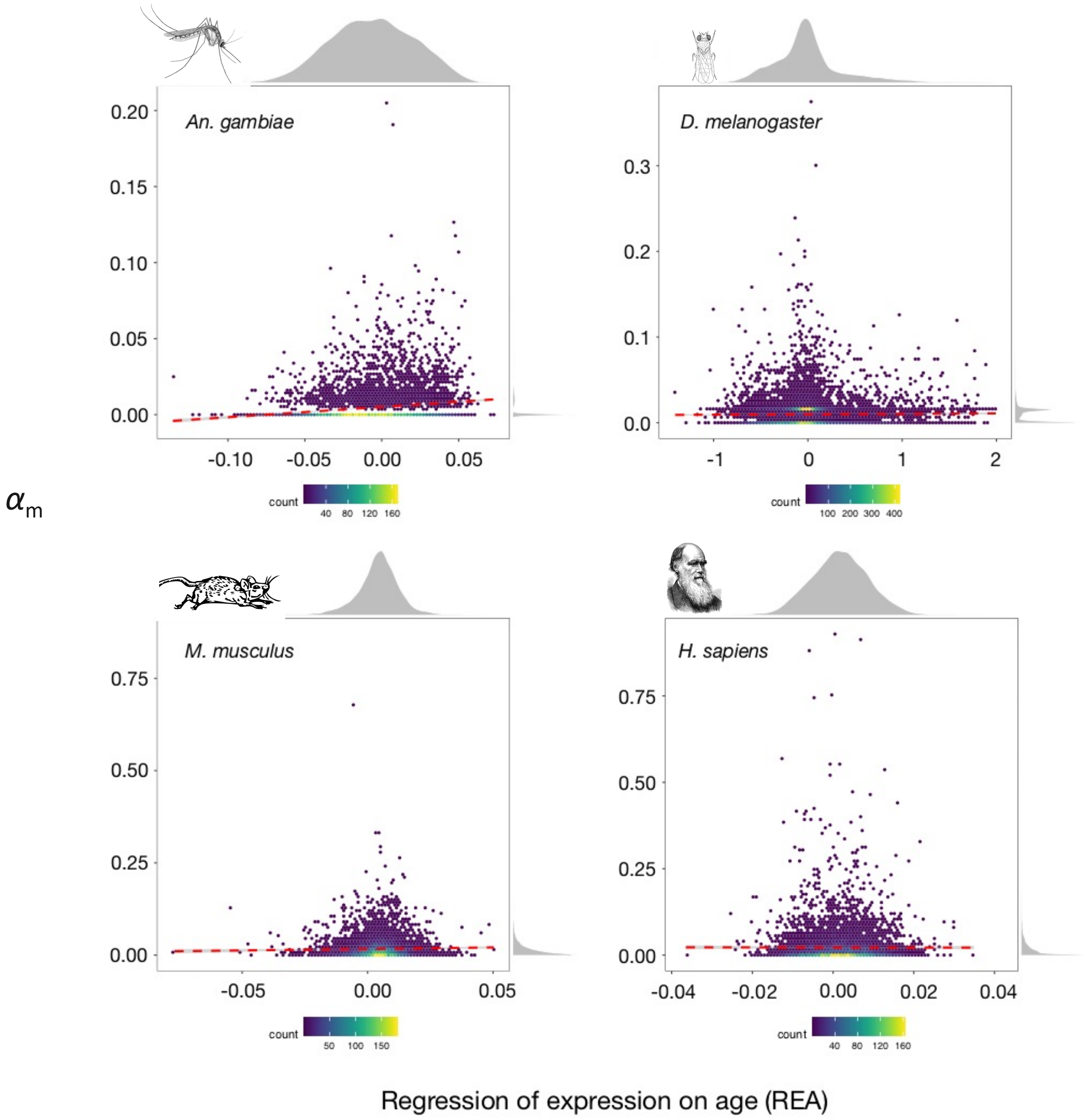

Supplemental Fig. S6: Late genes fix beneficial mutations more often. The fraction of nonsynonymous mutations that are beneficial and become fixed (α_m) is significantly greater in late-expressed than early-expressed genes in mosquitoes, mice, and humans. The dashed lines are least squares regressions, and the grey regions show the approximate 95% confidence intervals for the regressions. See also Fig. 6 and Table S1

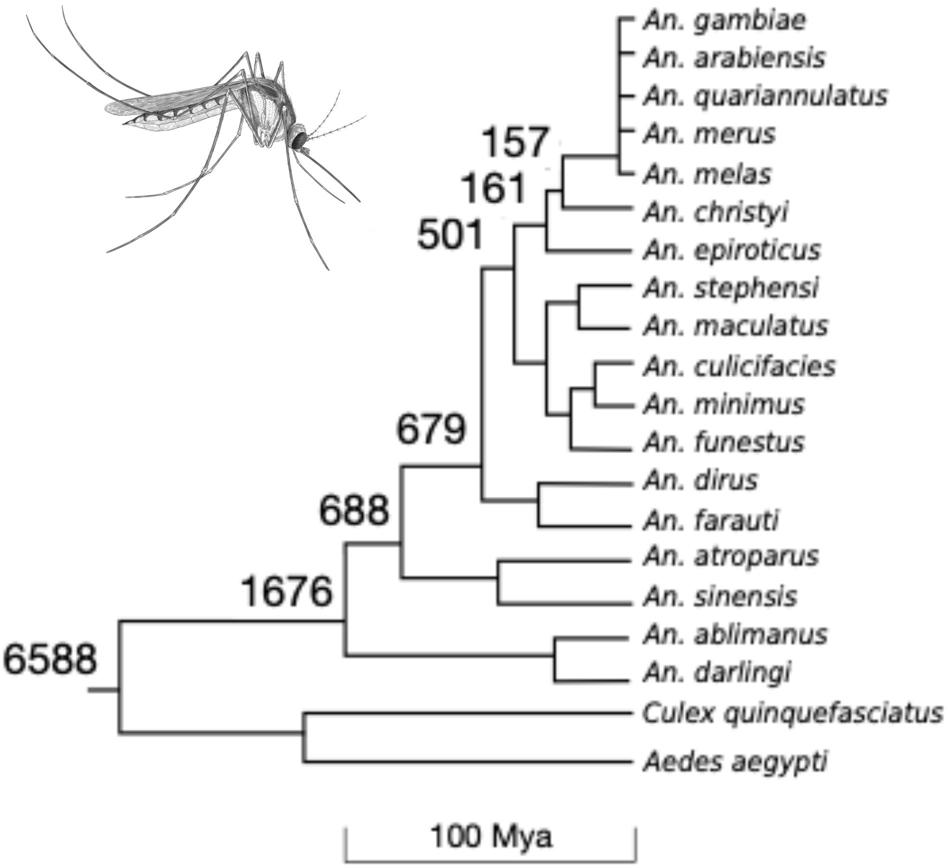

Supplemental Fig. S7: Ages of genes in the mosquito. Shown next to the nodes are the numbers of genes with an age equal to that of the node.

Supplementary Table 1. Spearman rank correlations between several molecular evolution statistics and REA. The last three statistics are comparisons of pairs of paralogs (see Table 2). Values in bold are significant at $p < 0.05$, and exact p values are given inside the parentheses (two-sided tests, not corrected for multiple tests).

Species	d_N/d_S	p_N/p_S	Age	$N_e\,s$	α_m	$\Delta d_N/d_S$	$\Delta p_N/p_S$	ΔAge
Mosquito	**0.10**	**0.16**	**-0.096**	**-0.16**	**0.15**	**0.10**	**0.18**	**-0.020**
	(2.4e-18)	(7.3e-46)	(1.2e-16)	(5.8e-46)	(1.9e-36)	(4.7e-94)	(1.8e-273)	(8.1e-05)
Fruit fly	**0.036**	**0.025**	**-0.048**	-0.0067	0.0038	**0.061**	0.017	**-0.032**
	(3.9e-03)	(5.8e-03)	(4.0e-08)	(0.47)	(0.68)	(2.7e-04)	(0.23)	(4.9e-02)
Mouse	**0.060**	**0.033**	**-0.039**	**-0.026**	**0.028**	**0.044**	**0.033**	**-0.068**
	(4.3e-05)	(6.3e-03)	(2.1e-03)	(3.5e-02)	(2.6e-02)	(3.8e-09)	(1.0e-06)	(2.2e-16)
Human	**0.032**	**0.020**	**-0.047**	**-0.032**	0.0071	**0.019**	0.017	**-0.021**
	(2.7e-03)	(8.9e-03)	(4.6e-09)	(4.8e-03)	(0.082)	(9.5e-05)	(0.099)	(1.9e-15)

Supplementary Table 2: Gene ontology enrichment analyses

Gene ontology terms that are most enriched in early- and late-expressed genes.

Terms are ranked by the probability of the observed enrichment. Statistically significant value terms (Q < 0.05) in bold.

Early expressed genes

Fly (77 significant terms)	
GO term	*Q*
**GO:0006397 mRNA processing**	3.76E-29
**GO:0006259 DNA metabolic process**	2.33E-27
**GO:0006412 translation**	3.86E-24
**GO:0005730 nucleolus**	5.89E-22
**GO:0051301 cell division**	8.46E-22
**GO:0140014 mitotic nuclear division**	1.79E-19
**GO:0042254 ribosome biogenesis**	5.06E-19
**GO:0005794 Golgi apparatus**	2.14E-17
**GO:0007059 chromosome segregation**	2.84E-16
**GO:0004386 helicase activity**	4.18E-16

Mosquito (57 significant terms)	
GO term	*Q*
**GO:0005739 mitochondrion**	9.45E-17
**GO:0006412 translation**	6.03E-12
**GO:0051276 chromosome organization**	3.94E-10
**GO:0005730 nucleolus**	1.45E-09
**GO:0042254 ribosome biogenesis**	3.21E-09
**GO:0005654 nucleoplasm**	5.24E-09
**GO:0005829 cytosol**	1.00E-08
**GO:0022618 ribonucleoprotein complex assembly**	1.41E-07
**GO:0065003 protein-containing complex assembly**	4.70E-07
**GO:0005840 ribosome**	7.64E-07

Mouse (11 significant terms)	
GO term	*Q*
**GO:0003735 structural constituent of ribosom**	1.82E-07
**GO:0005840 ribosome**	2.70E-07
**GO:0034655 nucleobase-containing compoun**	1.64E-02
**GO:0016887 ATPase activity**	1.64E-02
**GO:0003924 GTPase activity**	1.64E-02
**GO:0004386 helicase activity**	1.64E-02
**GO:0019843 rRNA binding**	2.57E-02
**GO:0051082 unfolded protein binding**	2.57E-02
**GO:0042254 ribosome biogenesis**	3.15E-02
**GO:0016779 nucleotidyltransferase activity**	3.63E-02

Human (1,692 significant terms)	
GO term	*Q*
**GO:0043312_neutrophil_degranulation**	1.79E-43
**GO:0016567_protein_ubiquitination**	6.15E-36
**GO:0043687_post-translational_protein_modification**	7.17E-30
**GO:0016032_viral_process**	9.63E-30
**GO:0051301_cell_division**	6.84E-29
**GO:0006468_protein_phosphorylation**	3.14E-28
**GO:0043066_negative_regulation_of_apoptotic_process**	7.03E-26
**GO:0045892_negative_regulation_of_transcription,_DNA-ter**	1.62E-25
**GO:0015031_protein_transport**	3.48E-25
**GO:0006511_ubiquitin-dependent_protein_catabolic_proces**	1.30E-24

Late expressed genes

Fly (0 significant terms)

GO term	Q
GO:0030312 external encapsulating structure	1.00
GO:0071554 cell wall organization or biogenesis	1.00
GO:0016798 hydrolase activity, acting on glycos	1.00
GO:0031012 extracellular matrix	1.00
GO:0030198 extracellular matrix organization	1.00
GO:0003013 circulatory system process	1.00
GO:0044403 symbiont process	1.00
GO:0019748 secondary metabolic process	1.00
GO:0005811 lipid droplet	1.00
GO:0016810 hydrolase activity, acting on carbon	1.00

Mosquito (4 significant terms)

GO term	Q
**GO:0050877 nervous system process**	1.54E-16
**GO:0005615 extracellular space**	5.96E-04
**GO:0005929 cilium**	2.39E-03
**GO:0031012 extracellular matrix**	2.39E-03
GO:0034330 cell junction organization	5.97E-01
GO:0000003 reproduction	5.97E-01
GO:0007267 cell-cell signaling	6.41E-01
GO:0007155 cell adhesion	1.00E+00
GO:0016798 hydrolase activity, acting on glycosyl bonds	1.00E+00
GO:0005811 lipid droplet	1.00E+00

Mouse (0 significant terms)

GO term	Q
GO:0016798 hydrolase activity, acting on glycos	0.670
GO:0007005 mitochondrion organization	0.670
GO:0005764 lysosome	0.670
GO:0016746 transferase activity, transferring acyl	0.670
GO:0016874 ligase activity	0.670
GO:0006399 tRNA metabolic process	0.670
GO:0006520 cellular amino acid metabolic proce	0.681
GO:0016757 transferase activity, transferring gly	0.681
GO:0005773 vacuole	0.681
GO:0006914 autophagy	0.741

Human (1 significant term)

GO term	Q
**GO:0050911_detection_of_chemical_stimulus_involved_in_s**	6.08E-05
GO:0052697_xenobiotic_glucuronidation	1
GO:0052695_cellular_glucuronidation	1
GO:0035195_gene_silencing_by_miRNA	1
GO:0007608_sensory_perception_of_smell	1
GO:0042573_retinoic_acid_metabolic_process	1
GO:0031424_keratinization	1
GO:0002323_natural_killer_cell_activation_involved_in_immun	1
GO:0050713_negative_regulation_of_interleukin-1_beta_secretio	1
GO:0048665_neuron_fate_specification	1

Supplementary Table 3. ANOVA results for effects of sex linkage and the regression of expression on age (REA) molecular evolution statistics.

*: $p < 0.05$. **: $p < 0.01$. ***: $p < 0.001$.

π

Species	R^2	Source	df	SS	F	η^2	p
Mosquito	$R^2 = 0.07$	Sex Chr.(S)	1	0.006889	432.7	0.0395	2e-16***
		REA (R)	1	0.005086	319.5	0.0292	2e-16***
		SxR	1	0.000073	4.6	0.0004	0.033*
		Error		0.162413			
		Total		0.174461			
Fly	$R^2 = 0.04$	Sex Chr.	1	0.002872	500.8	0.0330	4.4e-109***
		REA	1	0.000844	147.3	0.0097	1.0e-33***
		SxR	1	0.000047	8.2	0.0005	4.2e-03**
		Error		0.083152			
		Total		0.086915			
Mouse	$R^2 = 0.04$	Sex Chr.	1	0.0002907	265.6	0.0370	1.3e-58***
		REA	1	0.0000124	11.3	0.0016	7.8e-04***
		SxR	1	0.0000006	0.6	0.0001	0.44
		Error		0.0075457			
		Total		0.0078494			
Human	$R^2 = 0.004$	Sex Chr.	1	0.00000039	19.8	0.0022	8.6e-06***
		REA	1	0.00000032	16.1	0.0018	6.0e-05***
		SxR	1	0.00000001	0.3	0.0000	0.58
		Error		0.00017322			
		Total		0.000173929			

dN/dS

Species	R^2	Source	df	SS	F	η^2	p
Mosquito	$R^2 = 0.04$	Sex Chr.	1	0.22	11.3	0.0014	7.6e-04***
		REA	1	6.689	344.1	0.0419	3.2e-75***
		SxR	1	0	0.0	0.0000	0.96
		Error		152.871			
		Total		159.78			
Fly	$R^2 = 0.003$	Sex Chr.	1	0.0101	4.9	0.0008	0.026*
		REA	1	0.0248	12.2	0.0019	4.9e-04***
		SxR	1	0.0079	3.9	0.0006	0.049*
		Error		13.3111			
		Total		13.3539			
Mouse	$R^2 = 0.003$	Sex Chr.	1	0.165	7.1	0.0015	5.8e-03**
		REA	1	0.144	6.2	0.0013	1.4e-02*
		SxR	1	0.051	2.2	0.0005	0.16
		Error		110.158			
		Total		110.518			
Human	$R^2 = 0.0007$	Sex Chr.	1	0.02	0.1	0.0000	0.75
		REA	1	0.98	5.0	0.0011	0.025*
		SxR	1	0.28	1.4	0.0003	0.24
		Error		920.86			
		Total		922.14			

Gene Age

Species	R^2	Source	df	SS	F	η^2	p
Mosquito	$R^2 = 0.04$	Sex Chr.	1	36267	9.7	0.0008	1.9e-03***
		REA	1	1604534	427.6	0.0362	2.8e-93***
		SxR	1	6129	1.6	0.0001	0.20
		Error		42706745			
		Total		44353675			
Fly	$R^2 = 0.002$	Sex Chr.	1	526	7.0	0.0005	8.3e-03**
		REA	1	1696	22.4	0.0017	2.2e-06***
		SxR	1	91	1.2	0.0001	0.27
		Error		1003837			
		Total		1006150			
Mouse	$R^2 = 0.007$	Sex Chr.	1	310678	42.4	0.0066	6.1e-09***
		REA	1	56144	7.7	0.0012	1.7e-03**
		SxR	1	9887	1.3	0.0002	0.41
		Error		47023207			
		Total		47399916			
Human	$R^2 = 0.008$	Sex Chr.	1	1742803	113.5	0.0069	2.0e-26***
		REA	1	374974	24.4	0.0015	7.9e-09***
		SxR	1	17508	1.1	0.0001	0.29
		Error		249147164			
		Total		251282449			

Number of Paralogues

Species	R^2	Source	df	SS	F	η^2	p
Mosquito	$R^2 = 0.04$	Sex Chr.	1	2230	25.8	0.0022	7.6e-07***
		REA	1	37239	431.7	0.0362	4.6e-66***
		SxR	1	1655	19.2	0.0016	1.4e-08***
		Error		988036			
		Total		1029160			
Fly	$R^2 = 0.007$	Sex Chr.	1	1377	34.7	0.0023	4.0e-09***
		REA	1	2757	69.4	0.0047	8.6e-17***
		SxR	1	1	0.0	0.0000	0.87
		Error		584096			
		Total		588231			
Mouse	$R^2 = 0.002$	Sex Chr.	1	470	12.3	0.0018	4.6e-04***
		REA	1	214	5.6	0.0008	1.8e-02*
		SxR	1	58	1.5	0.0002	0.22
		Error		265291			
		Total		266033			
Human	$R^2 = 0.008$	Sex Chr.	1	769	22.6	0.0013	2.0e-06***
		REA	1	3962	116.7	0.0065	4.0e-27***
		SxR	1	29	0.9	0.0000	0.34
		Error		608561			
		Total		613321			
