# Supplementary material for: Molecular evolution and the decline of purifying selection with age
Source: Nat Commun. 2021 May 11;12:2657. doi: 10.1038/s41467-021-22981-9 (PMC8113359; doi:10.1038/s41467-021-22981-9)
Supplement: Supplementary file 3 — Description of Additional Supplementary Files [file 41467_2021_22981_MOESM3_ESM.pdf]

Description of Additional Supplementary Files

File Name: Supplementary Data 1
Description: The values for the regression of expression on age (REA) and gene age for genes in the mosquito *An. gambiae*.
