# Supplementary material for: Molecular evolution and the decline of purifying selection with age
Source: Nat Commun. 2021 May 11;12:2657. doi: 10.1038/s41467-021-22981-9 (PMC8113359; doi:10.1038/s41467-021-22981-9)
Supplement: Supplementary file 5 — Reporting Summary [file 41467_2021_22981_MOESM5_ESM.pdf]

nature research

Corresponding author(s): Mark Kirkpatrick

Last updated by author(s): 3/26/2021

Reporting Summary

Nature Research wishes to improve the reproducibility of the work that we publish. This form provides structure for consistency and transparency in reporting. For further information on Nature Research policies, see our Editorial Policies and the Editorial Policy Checklist.

Statistics

For all statistical analyses, confirm that the following items are present in the figure legend, table legend, main text, or Methods section.

n/a	Confirmed	
☐	☒	The exact sample size (n) for each experimental group/condition, given as a discrete number and unit of measurement
☐	☒	A statement on whether measurements were taken from distinct samples or whether the same sample was measured repeatedly
☐	☒	The statistical test(s) used AND whether they are one- or two-sided<br>*Only common tests should be described solely by name; describe more complex techniques in the Methods section.*
☐	☒	A description of all covariates tested
☐	☒	A description of any assumptions or corrections, such as tests of normality and adjustment for multiple comparisons
☐	☒	A full description of the statistical parameters including central tendency (e.g. means) or other basic estimates (e.g. regression coefficient) AND variation (e.g. standard deviation) or associated estimates of uncertainty (e.g. confidence intervals)
☐	☒	For null hypothesis testing, the test statistic (e.g. F, t, r) with confidence intervals, effect sizes, degrees of freedom and P value noted<br>*Give P values as exact values whenever suitable.*
☒	☐	For Bayesian analysis, information on the choice of priors and Markov chain Monte Carlo settings
☐	☒	For hierarchical and complex designs, identification of the appropriate level for tests and full reporting of outcomes
☐	☒	Estimates of effect sizes (e.g. Cohen's d, Pearson's r), indicating how they were calculated

Our web collection on statistics for biologists contains articles on many of the points above.

Software and code

Policy information about availability of computer code

Data collection	Almost all data were obtained from publications. The only new data are for gene expression in Anopheles mosquitoes. Nimblegen microarray data were processed using NIMBLESCAN v2.5 software (Roche NimbleGen). These data were filtered using the pipelines and software versions described in refs. 21 and 22 .
Data analysis	R v4.0.3<br>Bioconductor v3.11<br>gage v2.38.3<br>ggExtra v0.9<br>ggplot2 v3.3.3<br>ggpubr v0.4.0<br>lme4 v1.1-26<br>nlme v3.1-152<br>oligo v1.52.1<br>PopGenome v2.7.5<br>viridis v0.5.1

For manuscripts utilizing custom algorithms or software that are central to the research but not yet described in published literature, software must be made available to editors and reviewers. We strongly encourage code deposition in a community repository (e.g. GitHub). See the Nature Research guidelines for submitting code & software for further information.

Data

Policy information about availability of data

All manuscripts must include a data availability statement. This statement should provide the following information, where applicable:

- Accession codes, unique identifiers, or web links for publicly available datasets
- A list of figures that have associated raw data
- A description of any restrictions on data availability

The gene expression data for An. gambiae can be found in the ArrayExpress database under accession numbers E-MTAB-10110 and E-MTAB-2463. The gene dating results and REA coefficients for An. gambiae are given in Supplementary Data 1. All other data are available in the references cited in the Methods.

Field-specific reporting

Please select the one below that is the best fit for your research. If you are not sure, read the appropriate sections before making your selection.

- [x] Life sciences
- [ ] Behavioural & social sciences
- [ ] Ecological, evolutionary & environmental sciences

For a reference copy of the document with all sections, see nature.com/documents/nr-reporting-summary-flat.pdf

Life sciences study design

All studies must disclose on these points even when the disclosure is negative.

Sample size	For the mosquito expression study, there are 15 samples (5 time points, and 3 replicates each), and each replicate containing pooled whole body RNA of 20 to 30 individuals (ms. line 243). The number of replicates were decided based on previous published research on the same species and on the same microarray platform.
Data exclusions	No data exclusion
Replication	Three replicates
Randomization	For each replicate, individuals were randomly selected from a total of about 1,000 insects .
Blinding	No blinding. No bias can be introduced in data collection and processing by knowing the labels of the samples.

Reporting for specific materials, systems and methods

We require information from authors about some types of materials, experimental systems and methods used in many studies. Here, indicate whether each material, system or method listed is relevant to your study. If you are not sure if a list item applies to your research, read the appropriate section before selecting a response.

Materials & experimental systems

n/a	Involved in the study
x	Antibodies
x	Eukaryotic cell lines
x	Palaeontology and archaeology
	x Animals and other organisms
x	Human research participants
x	Clinical data
x	Dual use research of concern

Methods

n/a	Involved in the study
x	ChIP-seq
x	Flow cytometry
x	MRI-based neuroimaging

Animals and other organisms

Policy information about studies involving animals; ARRIVE guidelines recommended for reporting animal research

Laboratory animals	Malaria mosquito (Anopheles gambiae) adult females, one week to five weeks old
Wild animals	None.
Field-collected samples	None.
Ethics oversight	Not required for mosquitoes.

Note that full information on the approval of the study protocol must also be provided in the manuscript.
